# Eukaryote-wide sequence analysis of mitochondrial *β*-barrel outer membrane proteins

**DOI:** 10.1186/1471-2164-12-79

**Published:** 2011-01-28

**Authors:** Kenichiro Imai, Naoya Fujita, M Michael Gromiha, Paul Horton

**Affiliations:** 1 AIST, Computational Biology Research Center, Tokyo, Aomi, 135-0064, Japan; 2 Japan Society for the Promotion of Science, Tokyo, Chiyoda, 102-8472, Japan; 3 Taiho Pharmaceutical Company, Ibaraki, Tsukuba, 300-2611, Japan

## Abstract

**Background:**

The outer membranes of mitochondria are thought to be homologous to the outer membranes of Gram negative bacteria, which contain 100's of distinct families of *β*-barrel membrane proteins (BOMPs) often forming channels for transport of nutrients or drugs. However, only four families of mitochondrial BOMPs (MBOMPs) have been confirmed to date. Although estimates as high as 100 have been made in the past, the number of yet undiscovered MBOMPs is an open question. Fortunately, the recent discovery of a membrane integration signal (the *β*-signal) for MBOMPs gave us an opportunity to look for undiscovered MBOMPs.

**Results:**

We present the results of a comprehensive survey of eukaryotic protein sequences intended to identify new MBOMPs. Our search employs recent results on *β*-signals as well as structural information and a novel BOMP predictor trained on both bacterial and mitochondrial BOMPs. Our principal finding is circumstantial evidence suggesting that few MBOMPs remain to be discovered, if one assumes that, like known MBOMPs, novel MBOMPs will be monomeric and *β*-signal dependent. In addition to this, our analysis of MBOMP homologs reveals some exceptions to the current model of the *β*-signal, but confirms its consistent presence in the C-terminal region of MBOMP proteins. We also report a *β*-signal independent search for MBOMPs against the yeast and Arabidopsis proteomes. We find no good candidates MBOMPs in yeast but the Arabidopsis results are less conclusive.

**Conclusions:**

Our results suggest there are no remaining MBOMPs left to discover in yeast; and if one assumes all MBOMPs are *β*-signal dependent, few MBOMP families remain undiscovered in any sequenced organism.

## Background

The outer membrane of mitochondria and chloroplasts as well as Gram-negative bacteria, their evolutionary cousins, harbor proteins of *β*-barrel structure, known as *β*-Barrel Outer Membrane Proteins (BOMPs). The current version of the endosymbiotic theory of mitochondria origin [1] suggests that mitochondria descend from an *α*-proteobacteria, possibly similar to Rickettsiales, an order of intracellular parasites [2]. BOMPs are predicted to occupy about 3% of the proteomes of Rickettsiales [3]. Thus, unless Rickettsiales has gained many MBOMPs since its divergence from mitochondria, the *α*-proteobacteria ancestor of mitochondria might be expected to have possessed about 40 BOMPs. Currently four families of mitochodrial BOMPs (MBOMPs) have been identified: Tom40, Sam50 (Tob55), VDAC and Mdm10 [4]. These MBOMPs perform important functions in mitochondria. Tom40 is required for the import of mitochondrial precursor proteins into mitochondria, as it forms the import pore of the translocase of the outer membrane (TOM) complex [5,6]. Sam50 is the central component of the sorting and assembly machinery (SAM) complex and promotes the integration of proteins into the outer membrane [7-9]. Notably, only Sam50 shows clear sequence homology with non-mitochondrial proteins: Omp85 in bacterial and Toc75-V in chloroplasts [10-12]. Both Tom40 and Sam50 are essential for yeast cell viability [5,6,9]. The VDAC family serves as the diffusion pore for small molecules entering or leaving the mitochondria [13,14]. VDAC is also thought to contribute to membrane permeability in mitochondrial induced apoptosis [15,16] and is promising as a drug target because of its permeability [17]. In yeast, Mdm10 is required for mitochondrial morphology and dynamics [18]. Homologs of Mdm10 have not been reported in mammals. Mdm10 is reported to be part of the SAM complex and have a role in the biogenesis of MBOMPs [19,20]. Recent studies have shown that Mdm10 are members of the ER-mitochondria tethering complex [21].

Based on experimental evidence [22], yeast Mmm2 (Mdm34), was once considered to be an MBOMP, and in our previous work [23] we treated it as such. However, recently pairwise Hidden Markov Model (HMM) comparison has revealed that the SMP domain found in the N-terminal region of Mmm2, belongs to the TULIP superfamily of lipid/hydrophobic ligand-binding domains containing members with known (non-BOMP) structure [24].

As reviewed in [25,26], precursors of MBOMPs are synthesized in the cytosol without a classical N-terminal matrix targeting signal (MTS) or other known targeting signals, but are nonetheless imported across the outer membrane by the TOM complex. In the inter-membrane space, small Tim proteins then escort them to the SAM complex which inserts and assembles them. In spite of significant progress towards the identification of the components involved in the biogenesis of MBOMPs, the import and insertion signal of MBOMPs are still not clear. The first tertiary structure of an MBOMP, human VDAC-1, was recently solved [27-29]. Interestingly, VDAC-1 consists of 19 transmembrane *β*-strands, unlike all known bacterial BOMP (BBOMP) structures, which have even number of *β*-strands (but see [30] for an alternative interpretation). There is some sequence similarity between the VDAC family and Tom40 families. The structure of the C-terminal part of Tom40 is thought to be similar to VDAC-1 [27,31]. The number of MBOMP families is not known. Many BOMPs have been identified in bacteria, but ony four families have been found to date in mitochondria. Of course, some MBOMP families may remain undiscovered, but it is difficult to experimentally screen for MBOMPs, even in model organisms. Zahedi et al. [32] report a proteomics study of the mitochondrial outer membrane in yeast which detected 112 proteins, including both MBOMPs and *α*-helical proteins. Burri et al. [33] found that 11 proteins precipitate out of a fraction expected to contain MBOMPs and lipid-modified proteins. Neither of these results are specific to MBOMPs.

Searching for new MBOMPs by computational analysis has been difficult as well, due to the extreme divergence in sequence and structure between MBOMPs and their bacterial homologs (or analogs). Recent bioinformatic developments include an analysis using a homology detection method based on transitive sequence similarity search, in which similarity was measured by pairwise HMM profile comparison [34]. In that study the authors identified Sam50, VDAC and Tom40 as BBOMP homologs, but found no promising novel MBOMPs in the yeast or human proteome.

However, recent experimental studies have provided useful information to search for novel MBOMPs by sequence analysis: the discovery of the *β*-signal [35], a C-terminal motif, which is proposed to be the insertion signal of MBOMPs into the outer membrane; the determination of the first tertiary structure of VDAC-1 [27-29]; and the import analysis of BBOMPs in yeast [36]. In particular, the identification of the *β*-signal gave us the opportunity to search for novel candidates of MBOMPs by bioinformatics analysis. In preliminary work [23], we used the presence of a conserved *β*-signal in mitochondrial proteins to search for new candidate MBOMPs and came to the conclusion that probably very few MBOMPs remain undiscovered.

However, for several reasons, our preliminary study could not be considered conclusive: 1) we limited our search to proteins annotated by Uniprot [37] or Gene Ontology [38] as having possible mitochondria localization - but uncharacterized proteins might lack that annotation and 2) we limited the search for *β*-signals to the C-terminal 40 residues - but MBOMPs having an internal *β*-signal might exist. Moreover, recent experiments expressing BBOMPs in yeast [36], and results we report here on presumed homologs of known MBOMPs, call into question the necessity of the *β*-signal for all MBOMPs.

In this paper, we describe an expanded search which addresses those limitations. For this search we developed a new machine learning based predictor, trained on both bacterial and mitochondrial proteins, for the prediction of *β*-barrel membrane proteins based on sequence features designed to reflect structural constraints and specific sorting signals that MBOMPs are expected to lack. We report the results of a comprehensive search of known Eukaryotic protein sequences employing our new predictor, conserved C-terminal or internal *β*-signals, secondary structure prediction, and literature search. We also use our new predictor to conduct a *β*-signal independent search against the yeast and Arabidopsis proteomes. We found no new candidates in yeast, while in Arabidopsis several uncharacterized proteins met the search criteria, and one seems potentially promising.

We conclude that there probably are no new MBOMPs to be found in yeast and maybe very few MBOMPs remaining to be found at all, although we must admit that if *β*-signal independent or multimeric MBOMPs exist, our search might miss them.

Since MBOMPs are at the core of all known mitochondria outer membrane channels, our results suggest the possibility that the interface between the mitochondria and the cytosol is largely realized by a handful of protein families.

## Results and Discussion

### Conservation of *β*-signal

**Figure 1:**
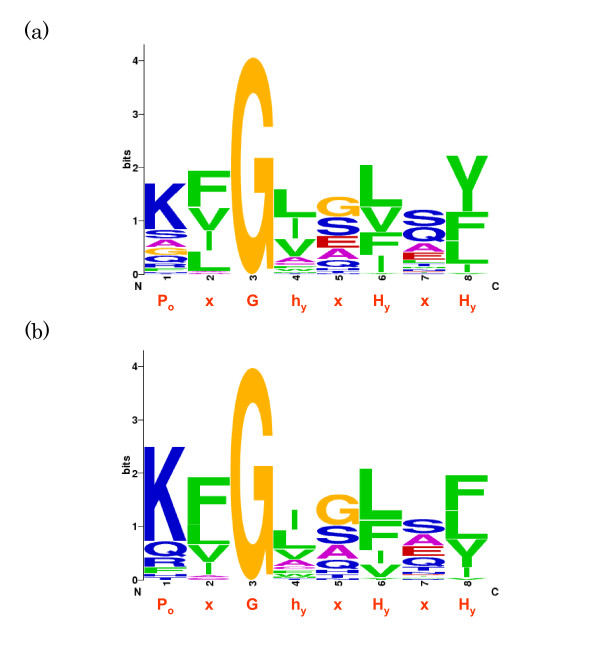
**Sequence logos of proposed *β*-signal regions of combined ortholog sets**. The upper figure is computed from all homologs in Uniprot and the bottom from the subset of those with confirmed expression.

Kutik et al. [35] proposed the "*β*-signal", with the motif P_o_xGxxH_y_xH_y _(P_o_, polar residue; G, glycine; H_y_, large hydrophobic residue; x, any residue) occurring near the end of the most C-terminal *β*-strand. Figure 1 shows the sequence logo [39,40] of 41 and 57 unique *β*-signal motif sequences; obtained from multiple sequence alignments of 46 MBOMP homologs with confirmed expression, and 70 MBOMPs including proteins whose existence is only inferred by homology, respectively.

#### β-signal position

**Figure 2:**
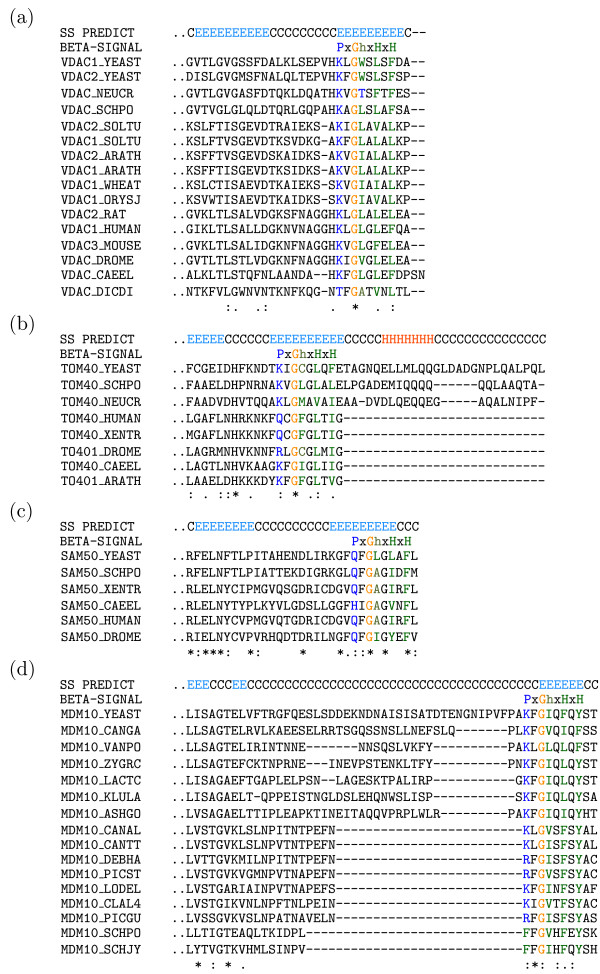
**Conserved *β*-signals and secondary structure prediction of yeast MBOMPs**. In the top track, H, E, and C represented *α*-helix, *β*-strand and coil, as predicted by PSIPRED or from the experimentally determined structure in the case of VDAC (PDB:2K4T).

For each known MBOMP family (Tom40, Sam50, VDAC and Mdm10), a well conserved *β*-signal motif match occurs near the C-terminus around or near the final known or predicted *β*-strand (Figure 2).

#### β-signal consensus

The increase of available protein sequences since our preliminary study [23], allowed us to refine the *β*-signal motif somewhat. As indicated in Table 1 which shows the frequency of amino acid groups in the *β*-signal motif match of 70 putative MBOMP homolog sequences, the first residue of the *β*-signal motif is usually polar, but no negatively charged residues are observed. The residue following glycine (the 4th position) is almost always hydrophobic. Thus we proposed [23] (and Kutik et al. further clarified [41]) a slightly refined motif pattern: P_o_xGh_y_xH_y_xH_y _(P_o _non-negatively charged polar residue, G glycine, H_y _large hydrophobic residue, h_y _hydrophobic residue including Ala and Cys, x any residue). As can be seen in Table 1 and the sequence logos of Figure 1, the second position is nearly always a hydrophobic residue. Thus P_o_H_y_Gh_y_xH_y_xH_y _could also be considered as an alternative consensus sequence. Moreover the other "x" positions also clearly show non-random residue preferences, for example, smaller residues such as glycine, serine, alanine are abundant, however the commonly observed residues do not all share similar properties in terms of hydrophobicity; and because the sequences used are not all mutually independent, the column heights in their sequence logo do not necessarily indicate statistical significance (see methods for details).

**Table 1:** The frequency of amino acid groups of *β*-signal motif sequences from 70 MBOMP homologs.

	Motif position								
	**Background**	**P_*o*_**	**X**	**G**	**h_*y*_**	**X**	**H_*y*_**	**X**	**H_*y*_**

Large hydrophobic (L, I, V, F, M, W, Y)	0.32	0.03	**0.96**	0.00	**0.89**	0.00	**1.00**	0.16	**1.00**
Small hydrophobic (A, C)	0.08	0.07	0.04	0.00	**0.10**	0.11	0.00	0.11	0.00
Glycine	0.07	0.16	0.00	**1.00**	0.00	0.24	0.00	0.00	0.00
Non-negatively charged polar (K, R, H, S, T, N, Q)	0.39	**0.74**	0.00	0.00	0.01	0.50	0.00	0.61	0.00
Negatively charged (D, E)	0.09	0.00	0.00	0.00	0.00	0.14	0.00	0.11	0.00
Proline	0.05	0.00	0.00	0.00	0.00	0.00	0.00	0.00	0.00

The numbers in parentheses are frequencies of each amino acid group in each position of the *β*-signal in 70 MBOMP sequences. "Background" is the overall frequency of each amino acid group in the entire set of sequences.

In any case, the *β*-signal region of MBOMPs, for the most part including the "x" positions as well, has a dyad periodicity of hydrophobic residues, and is devoid of the secondary structure breaker proline; as would be expected for a *β*-strand in which one side faces the membrane and the other an aqueous pore [42]. This is consistent with the original proposal of Kutik et al. [35] and the recently determined micelle structure of VDAC [27,28], in which the *β*-signal occurs in the final *β*-strand.

#### β-signal motif match occurrence frequency

The *β*-signal was proposed as a signal found near the end of the barrel region, and known MBOMPs generally have *β*-signal motif matches near their C-terminal. However, except for the possibly invariant glycine, the *β*-signal allows for much variation. Moreover, the *β*-signal motif is consistent with the general pattern of alternating hydrophobic and hydrophilic residues expected from any membrane barrel *β*-strand. The lack of proline residues is also expected from secondary structure. Thus one may wonder if matches to the *β*-signal motif would be expected to occur by chance in most barrel strands. To investigate this we assumed a statistical null model, in which the frequency of *β*-signal matches is the same in the length 40 C-terminal region as it is in a randomly chosen length 40 region not overlapping with the length 40 C-terminal region. A drawback of this model is that ideally we should exclude the non-barrel portion of MBOMP from consideration but we did not do this. However, the structure of VDAC [27-29] suggests that most of its sequence, at least, is part of its *β*-barrel.

In any case, we computed the probability of this null model for VDAC, Tom40, Sam50 and Mdm10 (Table 2), using ten versions of the *β*-signal motif. The most significantly enriched pattern for Sam50 is ${\text{P}}_{\text{o}}{\text{H}}_{\text{y}}{\text{Gh}}_{\text{y}}\bar{H_{y}}\;{\text{H}}_{\text{y}}{\text{xH}}_{\text{y}}$, where $\bar{H_{y}}$ denotes any residue except [LIVFMWY]. The *β*-signal of each of 5 Sam50 homologs matches this pattern, but no other matches are found within those sequence. For VDAC P_o_H_y_Gh_y_xH_y_xH_y _and ${\text{P}}_{\text{o}}{\text{H}}_{\text{y}}{\text{Gh}}_{\text{y}}\bar{H_{y}}\;{\text{H}}_{\text{y}}{\text{xH}}_{\text{y}}$; for Tom40, P_o_xGh_y_xH_y_xH_y_; and for Mdm10, $\bar{{\text{H}}_{\text{y}}}{\text{H}}_{\text{y}}{\text{Gh}}_{\text{y}}{\text{xH}}_{\text{y}}{\text{xH}}_{\text{y}}$ and $\bar{{\text{H}}_{\text{y}}}{\text{H}}_{\text{y}}{\text{Gh}}_{\text{y}}\bar{{\text{H}}_{\text{y}}}{\text{xH}}_{\text{y}}$ are the most enriched. Interestingly, for each of the four MBOMP families, the *β*-signal motif is still enriched even if the glycine is ignored. Before seeing this result, we were inclined to view the glycine as the heart of the *β*-signal and the other positions to perhaps simply reflecting secondary structure. However, patterns such as ${\text{P}}_{\text{o}}{\text{H}}_{\text{y}}{\text{xh}}_{\text{y}}\bar{{\text{H}}_{\text{y}}}{\text{H}}_{\text{y}}{\text{xH}}_{\text{y}}$, skipping the requirement for glycine in the third position, still attain *p*-values around 10^-3 ^or less; suggesting that the other positions may also contribute in a specific way to the recognition of the *β*-signal.

**Table 2:** Comparison of the frequency of *β*-signal motif matches in the C-terminal 40 residues compared with the rest of the sequence of MBOMPs

Motif pattern	VDAC	Tom40	Sam50	Mdm10
P_o _x G x x H_y_xH_y_	(4/4, 0.20, 1.6 × 10^-3^)	(5/5, 0.12, 2.5 × 10^-5^)	(5/5, 0.14, 5.4 × 10^-5^)	(6/8, 0.17, 4.9 × 10^-4^)
P_o _x Gh_y _x H_y_xH_y_	(4/4, 0.09, 6.6 × 10^-5^)	(5/5, 0.09, 5.9 × 10^-6^)	(5/5, 0.09, 5.9 × 10^-6^)	(6/8, 0.07, 2.9 × 10^-6^)
P_o _H_y_Gh_y _x H_y_xH_y_	(4/4, 0.07, 2.4 × 10^-5^)	(4/5, 0.07, 1.1 × 10^-4^)	(5/5, 0.02, 3.2 × 10^-9^)	(4/8, 0.04, 1.6 × 10^-4^)
P_o _H_y _xh_y _x H_y_xH_y_	(4/4, 0.25, 3.9 × 10^-3^)	(5/5, 0.17, 1.4 × 10^-4^)	(5/5, 0.12, 2.5 × 10^-5^)	(5/8, 0.12, 1.0 × 10^-3^)
${\text{P}}_{\text{o}}{\text{H}}_{\text{y}}{\text{Gh}}_{\text{y}}\bar{{\text{H}}_{\text{y}}}\;{\text{H}}_{\text{y}}{\text{xH}}_{\text{y}}$	(4/4, 0.07, 2.4 × 10^-5^)	(4/5, 0.07, 1.1 × 10^-4^)	(5/5, 0.00, 0.0)	(4/8, 0.04, 1.6 × 10^-4^)
${\text{P}}_{\text{o}}{\text{H}}_{\text{y}}{\text{xh}}_{\text{y}}\bar{{\text{H}}_{\text{y}}}{\text{H}}_{\text{y}}{\text{xH}}_{\text{y}}$	(4/4, 0.21, 1.9 × 10^-3^)	(5/5, 0.17, 1.4 × 10^-4^)	(5/5, 0.08, 3.3 × 10^-6^)	(5/8, 0.12, 1.0 × 10^-3^)
$\bar{{\text{H}}_{\text{y}}}{\text{H}}_{\text{y}}{\text{Gh}}_{\text{y}}{\text{ x H}}_{\text{y}}{\text{xH}}_{\text{y}}$	(4/4, 0.11, 1.5 × 10^-4^)	(4/5, 0.07, 1.1 × 10^-4^)	(5/5, 0.02, 3.2 × 10^-9^)	(7/8, 0.04, 1.3 × 10^-9^)
$\bar{{\text{H}}_{\text{y}}}{\text{H}}_{\text{y}}{\text{xh}}_{\text{y}}{\text{ x H}}_{\text{y}}{\text{xH}}_{\text{y}}$	(4/4, 0.37, 1.9 × 10^-2^)	(5/5, 0.23, 6.4 × 10^-4^)	(5/5, 0.22, 5.2 × 10^-4^)	(8/8, 0.18, 1.1 × 10^-6^)
$\bar{{\text{H}}_{\text{y}}}{\text{H}}_{\text{y}}{\text{Gh}}_{\text{y}}{\text{xH}}_{\text{y}}{\text{xH}}_{\text{y}}$	(4/4, 0.11, 1.5 × 10^-4^)	(4/5, 0.07, 1.1 × 10^-4^)	(5/5, 0.00, 0.0)	(7/8, 0.04, 1.3 × 10^-9^)
$\bar{{\text{H}}_{\text{y}}}{\text{H}}_{\text{y}}{\text{xh}}_{\text{y}}\bar{{\text{H}}_{\text{y}}}{\text{H}}_{\text{y}}{\text{xH}}_{\text{y}}$	(4/4, 0.33, 1.2 × 10^-2^)	(5/5, 0.23, 6.4 × 10^-4^)	(5/5, 0.20, 3.2 × 10^-4^)	(8/8, 0.16, 4.3 × 10^-7^)

Statistics on the frequency of matches in the C-terminal versus non-C-terminal part of MBOMPs families is shown for the 4 known MBOMPs and 10 variations of the *β*-signal motif. Each cell holds a triple: the fraction of homologs with C-termini which match the motif, the fraction of non-C-terminal length 40 substrings of the MBOMP sequences which match the motif, and a *p*-value. The *p*-value is computed with a binomial test, in which the non-C-terminal frequency (e.g. 0.2) is the probability of success and the C-terminal frequency (e.g 4/4) is the observed data.

Although these results are suggestive, we must warn the reader to interpret the *p*-values with caution. As mentioned above, one problem is the fact that some non-barrel regions are included in non-C-terminal statistics. Perhaps a more serious flaw is that our logic is somewhat circular, because the *β*-signal motif was partially derived from multiple sequence alignment of the C-terminal region of some of these MBOMP homologs (although also due to the results of mutational analysis experiments [35]). Finally the homolog sequences are not mutually independent (maximum pairwise identity is 40%), but the *p*-value computation assumes independence.

### Conserved *β*-signal based MBOMP search

**Figure 3:**
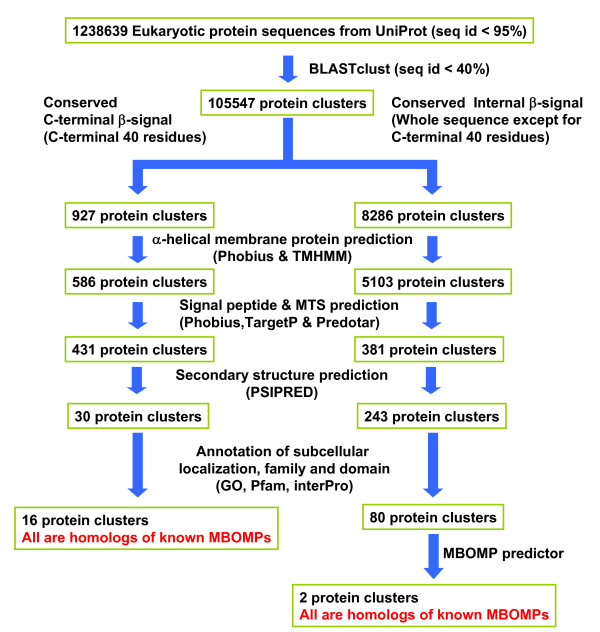
**Informatics pipeline for searching for novel MBOMP candidates**.

Our search started with 1,238,639 eukaryotic protein sequences from Uniprot version 15.1. We applied a simple filter to remove nearly identical sequences, clustered with blastclust at 40% identity and removed singleton clusters to obtain 105,547 homolog clusters. Figure 3 shows our search pipeline (see Methods for more details) and the number of candidates surviving each filter. 16 and 2 protein sequence clusters survived all steps of the C-terminal (C-terminal 40 residues region) and internal *β*-signal (all but C-terminal 40 residue region) search respectively.

Table 3 lists the representative proteins for each cluster. In Interpro [43], yeast VDAC and Tom40 are categorized as Porin, eukaryotic type family and Sam50 is annotated as having the bacterial surface antigen domain. Mdm10 belongs to the MDM10 family in Uniprot. All proteins in the list belong to the same family or share a characteristic domain with known or proposed yeast MBOMPs. Of the 18 (16+2) surviving representative proteins, we believe each are members of known MBOMP families: 9 Sam50, 4 Tom40, 3 VDAC and 2 Mdm10. The 9 proposed Sam50 homologs are annotated as having a bacterial surface antigen domain in their C-terminal regions, a characteristic shared by the MBOMP Sam50, the chloroplastic BOMP Toc75-V and bacterial Omp85. An N-terminal polypeptide transport-associated (POTRA) domain is also an important feature of these proteins. The tertiary structure of the POTRA domain of Omp85 revealed that Omp85 of *E*. coli has five POTRA domains which share the fishhook like fold consist of *β*1-*α*1-*α*2-*β*2-*β*3, where *α *and *β *denote *α *helices and *β *strands (in other bacterial species the number of POTRA domains is not necessarily five but can be between three (or even less) and seven.) [44,45]. Sam50 proteins are predicted to have one POTRA domain. Thus we compared the N-terminal region of the 9 proteins by multiple alignment and secondary structure predictions. The multiple alignment and secondary structure prediction suggest that these 9 proteins share one *β*1-*α*1-*α*2-*β*2-*β*3 pattern near their N-terminals (Additional file 1, Figure S1), although there were the differences in the length and composition of the predicted secondary structure elements. Thus we conclude that these 9 proteins are probably Sam50 homologs. 2 of the 16 representative proteins are the yeast and *Lodderomyces elongisporus *Mdm10 protein. The remaining 7 representatives are all listed as "Porin, eukaryotic type" in Interpro. One is yeast Tom40. A comparison of secondary structure prediction between yeast VDAC, Tom40, B8NA08, A8I528, B8BQH4, Q293I2 and Q7RE39 indicated that B8NA08, A8I528 and B8BQH4 belong to the VDAC family, while Q293I2 and Q7RE39 probably belong to the Tom40 family (data not shown). Finally, B7Z4T8 shares sequence similarity with Tom40 (Uniprot), although it is much shorter than other Tom40 proteins.

**Table 3:** List of identified protein clusters with conserved *β *-signal.

Representative protein	Number of cluster member	Organism	Protein length	Conserved motif position	Subcellular Localization	Family and domain
**C-terminal *β*-signal**						

SAM50-like protein CG7639 (Q9V784)	12	*D. melanogaster*	443	435-442	Mito OM (G)	SAM50/omp85 family (U), Bacterial surface antigen (I)
SAM complex subunit of the mitochondrial outer membrane, putative (B9WEF8)	6	*C. dubliniensis*	499	492-498	OM (G)	Bacterial surface antigen (I)
SAM50-like protein (A3LZ83)	6	*P. stipitis*	489	473-480	-	-
SAM50 (P53969)	5	*S. cerevisiae*	484	476-483	Mito OM (U, G)	SAM50/omp85 family (U), Bacterial surface antigen (I)
SAM50-like protein SpAC17C9.06 (Q10478)	2	*S. pombe*	475	467-474	Mito OM (G)	SAM50/omp85 family (U), Bacterial surface antigen (I)
SAM50-like protein gop-3 (P46576)	2	*C. elegans*	434	426-433	Mito OM (G)	SAM50/omp85 family (U), Bacterial surface antigen (I)
KLLA0E02223p (Q6CPU1)	2	*K. lactis*	480	472-479	OM (G)	Bacterial surface antigen (I)
Predicted cell surface protein homologous to bacterial outer membrane proteins (ISS) (Q017Y3)	2	*O. tauri*	521	508-515	OM (G)	Bacterial surface antigen (I)
TOM40 (P23644)	36	*S. cerevisiae*	387	352-359	Mito OM (U, G)	Tom40 family (U), Porin, eukaryotic type (I), Mitochondrial outer membrane translocase complex, subunit Tom40 (I)
GA18230 (Q293I2)	8	*D. pseudoobscura*	321	290-297	Mito OM (G)	Porin, eukaryotic type (I)
Outer mitochondrial membrane protein porin (B8NA08)	5	*A. flavus*	346	337-344	Mito OM (G)	Porin, eukaryotic type (I)
Voltage-dependent anion-selective channel protein (A8I528)	2	*C.reinhardtii*	276	268-275	OM (G)	Porin, eukaryotic type (I)
Predicted protein (B8BQH4)	2	*T. pseudonana*	268	261-268	OM (G)	Porin, eukaryotic type (I)
cDNA FLJ52528, highly similar to Protein TOMM40-like (B7Z4T8)	2	*H. sapiens*	210	202-209	OM (G)	Porin, eukaryotic type (I)
Mdm10 (P18409)	5	*S. cerevisiae*	493	484-491	Mito OM (U, G)	MDM10 family., Protein of unknown function DUF3722 (I)
Mdm10 (A5DUG6)	5	*L. elongisporus*	523	496-503	Mito OM (U, G)	MDM10 family., Protein of unknown function DUF3722 (I)
						
**Internal *β*-signal**						

Probable mitochondrial import receptor sub-unit tom40 homolog (Q7RE39)	10	*P. yoelii*	396	331-338	Mito OM (U, G)	Porin, eukaryotic type (I)
Putative uncharacterized protein (Q4CQ17)	5	*T. cruzi*	479	417-424	OM (G)	Bacterial surface antigen (I)

U, G and I in "Subcellular localization" and "Family and domain" represent the source of annotation from Uniprot, Gene ontology and InterPro, respectively

### Exceptions to the *β*-signal consensus motif

**Figure 4:**
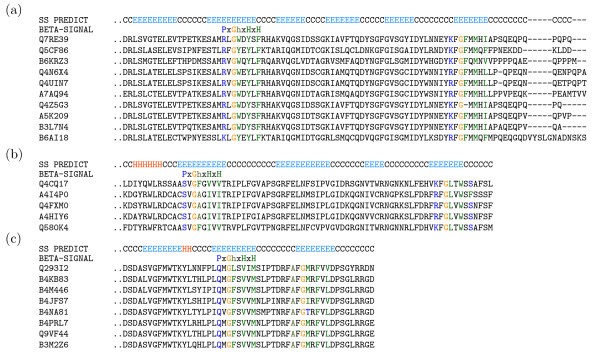
***β*-Signal matches not always in final predicted *β*-strand**. Protein clusters which match our automatic criteria of a conserved *β*-signal, but with the matches outside of the final predicted *β*-signal are shown. The top track indicates predicted *β *-strand (E), coil (C), or *α*-helix (H), by PSIPRED. The colored residues occur in full or partial matches to the *β*-signal motif.

As detailed above, we believe the two protein sequence clusters which passed the internal *β*-signal path of our search pipeline are homologs of known MBOMPs. However it is not clear that they really have internal *β*-signals. The *β*-signal motif matches of Q7RE39 and Q4CQ17 are not found in their final predicted *β*-strand (Figure 4). The proteins Q7RE39, Q4CQ17 and Q293I2 (detected in the C-terminal *β*-signal path of our pipeline) have conserved *β*-signal motif matches near their fifth, fourth and second predicted *β*-strands from their C-terminals, respectively. Focusing on the final predicted *β*-strand of these three proteins; Q4CQ17 and Q293I2 have partial *β*-signal motif matches such as KFGLTWSS and AFGMRFVV, while Q7RE39 has a match KFGFMMHI but one protein in its cluster, a putative Tom40 of *Plasmodium berghei *(Q4Z5G3), only matches (partially) when a gap is inserted after glycine (KFG-MMHI).

**Figure 5:**
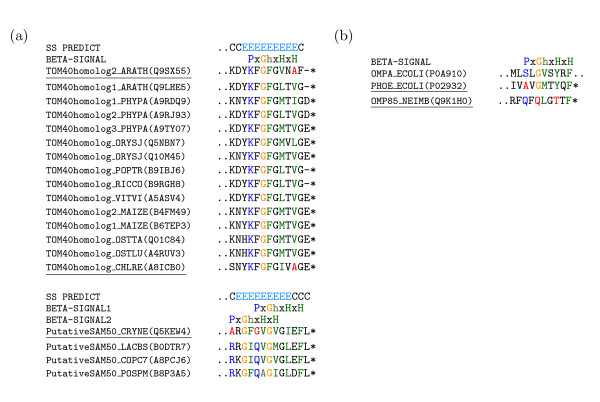
**Examples which don't match the ***β***-signal motif**. (a) Putative MBOMP homologs, which do not match the *β*-signal motif, and (b) BBOMPs sorted to the mitochondrial outer membrane when expressed in yeast are shown. The C-terminus is indicated with an asterisk.

Upon examining clusters which did not pass our conserved *β*-signal based search, we found some included homologs to known MBOMPs that do not possess perfect matches to the *β*-signal motif. Namely, the Tom40 homolog 2 of *Arabidopsis thaliana*, Tom40 of *Chlamydomonas reinhardtii*, and a putative SAM50 homolog of *Cryptococcus neoformans *(Figure 5). In that figure we also show the N-terminal regions of the BBOMPs: PhoE, OmpA (*Escherichia coli*) and Omp85 (*Neisseria meningitidis*) reported by Walther et al. [36] to be "correctly" integrated into the mitochondrial outer membrane when expressed in yeast. As those authors point out, PhoE and Omp85 do not have an appropriate match to the *β*-signal motif (Figure 5).

Moreover, in our analysis of 70 putative known MBOMP homologs, we found that 13 did not match the *β*-signal motif. For example, The *Neurospora crassa *VDAC sequence has threonine in the 4th position following the glycine and the putative *Schizosaccharomyces pombe *and *Schizosaccharomyces japonicus *Mdm10 *β*-signal sequences are FFGVHFEY and FFGIHFQY, with a phenylalanine instead of a polar residue in the first position (Figure 2). In Uniprot, the protein existence field of 11 of those is listed as "inferred by homology", but two exceptions are verified proteins: VDAC of N.*crassa *and Mdm10 of S.*pombe*.

### *β*-signal independent yeast MBOMP search

Despite allowing the possibility of internal *β*-signals, our conserved *β*-signal based search for MBOMPs failed to yield any promising new candidates. To make our search as comprehensive as possible, we also considered the possibility that some MBOMPs have no *β*-signal at all.

**Figure 6:**
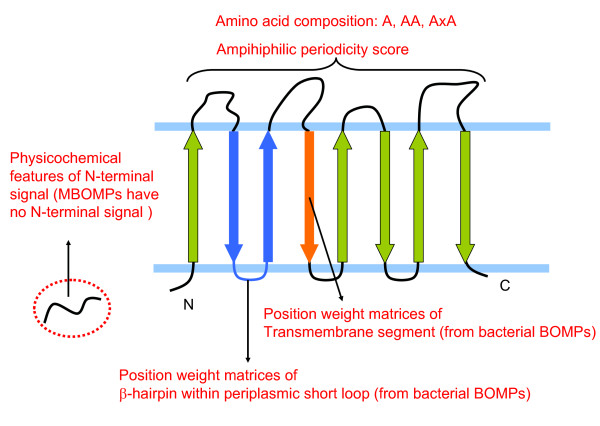
**Overview of features used for MBOMP prediction by SVM**.

We searched the yeast proteome (6470 yeast proteins obtained from Uniprot) for MBOMPs lacking *β*-signals by combining our new *β*-signal independent MBOMP prediction method combined with PSIPRED [46] secondary structure prediction and annotation based manual inspection. The features used by our new MBOMP predictor are: three types of amino acid composition; physicochemical features of the N-terminal sorting region, two amphiphilicity scores based on periodicity and position weight matrices of bacterial transmembrane *β*-strands and 2-strand *β*-hairpins (see Figure 6 and Methods for more details). Only a single sequence, that of the uncharacterized protein "YJL217W", passed our *β*-signal independent search against the yeast proteome.

#### Comparison to Proteomics Studies in Yeast

As mentioned in the background section, two experimental studies have tried to identify outer membrane proteins in yeast.

Zahedi et al. [32] report a proteomics study of the mitochondrial outer membrane in yeast which detected 112 proteins including all known MBOMPs. However their method cannot distinguish between MBOMPs, outer membrane *α*-helical proteins (common in mitochondria) and some peripheral membrane proteins. We find only 5 proteins in yeast (including two isoforms of VDAC) instead of 112, but this may be explained by the non-MBOMP proteins included in their list. In another experiment on yeast by Burri et al. [33], 11 proteins precipitated out of a Triton X-114 detergent phase expected to contain MBOMP or lipid-modified proteins. Two were determined to be the MBOMPs VDAC-1 and Tom40, and one, which the authors speculate may be lipid-modified, contained peptides derived by Xdj1. The only information given for the remaining eight is their molecular masses, which are different than known yeast MBOMPs. Without knowing the identity of these proteins we cannot give a specific analysis. One possibility is that those eight proteins are lipid-modified but not MBOMPs.

#### Is YJL217W an MBOMP?

Secondary structure prediction shows YJL217W is an all *β*-protein with a match to the *β*-signal motif around the most C-terminal predicted *β*-strand (Additional file 2, Figure S2(a)). (YJL217W was not considered in our conserved *β*-signal search because it did not cluster with any other sequences.) YJL217W has no known eukaryotic homologs. According to annotations of InterPro, YJL217W belongs to the DUF1349 family along with several hypothetical bacterial proteins based on sequence similarity; but the function of this family is unknown. YJL217W has no predicted signal peptide or matrix targeting signal, which is consistent with being an MBOMP. However, recently the structure of YJL217W was solved (PDBID:3O12, no published report)(Additional file 2, Figure S2(b)). According to this structure, YJL217W is not a *β*-barrel, but instead forms a *β*-sandwich structure like Concanavalin. Additionally, YJL217W was not identified as an mitochondrial outer membrane protein in the proteomics study of Zahedi et al. [32], and was found to have a cytosolic localization in a global analysis of protein localization in budding yeast [47]. Thus we do not think YJL217W is an MBOMP.

### *β*-signal independent Arabidopsis search

Yeast have relatively small proteomes and are evolutionarily very distant from plants and animals. This limits the scope of what we can conclude from the yeast *β*-signal independent search. Ideally we would like to perform a *β*-signal independent search on all Eukaryotic proteins, but unfortunately our pipeline partially depends on manual inspection of annotation and *ad hoc *analysis, so such a search would be infeasible in terms of both the amount of work entailed and the lack of experimental annotation for most species.

As a compromise, we chose to perform a *β*-signal independent search on the well annotated plant Arabidopsis. Plants contain mitochondria and chloroplasts, both of which are thought to descend from Gram negative bacteria and are known to have BOMPs in their outer membranes. Our MBOMP predictor was not trained on any chloroplastic BOMPs (CBOMPs), but to the extent that CBOMPs are similar to other BOMPs, we might expect our classifier to be able to detect them.

Several CBOMPs have been identified so far (five isoforms (I-V) of Toc75, OEP37, OEP24 and OEP21) [4,48]. For Arabidopsis, Uniprot lists three isoforms of Toc75 (III-V), OEP37, OEP24 and OEP21 and four MBOMPs were annotated (two homologs of Tom40 and two of VDAC). We applied our *β*-signal independent pipeline to the Arabidopsis proteome (35555 Arabidopsis proteins with less than 90% sequence identity downloaded from Uniprot). As in the yeast proteome analysis, we used our *β*-signal independent MBOMP prediction method combined with secondary structure prediction, *α*-helical membrane protein and signal peptide prediction, and finally manual inspection of annotations to find novel MBOMP (or CBOMP) candidates. 60 proteins passed the automated steps of our pipeline, but we rejected 22 of these based on domain and other annotation (Table 4). 13 of the remaining 38 proteins are annotated BOMPs, 4 MBOMPs (two Tom40 homolog and two VDAC) and 9 CBOMPs (Toc75-V/OEP80, OEP37, OEP24, OEP21, and five of their homologs). Another five proteins contain the Eukaryotic porin domain and four contain the Bacterial surface antigen (I) domain. This indicates that these proteins probably belong to the VDAC, Tom40, or the Sam50/Omp85 families, respectively. Most of the remaining 16 proteins have no annotation of functional domain or family. We attempted to gain some information by presenting these sequences to the BOMP predictor HHomp [34], and the sequence structure similarity detectors HHpred [49] and FORTE [50]. These predictors suggested that one of the proteins, Q9SXB7:At1g11320, has partial similarity to proteins with *β*-barrel structure. However no such similarity was detected for the remaining 15 proteins. Only one of these proteins, Q9M238, posesses an appropriately placed match to the *β*-signal, TLGYAFLV (it was not detected in our Eukaryotic-wide search, because its *β*-signal was not conserved in all members of its sequence cluster).

**Table 4:** List of identified proteins in our Arabidopsis proteome analysis.

Uniprot AC	Identification	Length	Highest score segment (score)	Domain and family in predicted region
MBOMP				
Q9SRH5	VDAC1	276	Whole sequence (0.989)	Porin, eukaryotic
Q9SMX3	VDAC2	274	Whole sequence (1.000)	Porin, eukaryotic
Q9FJX3	-	276	Whole sequence (0.989)	Porin, eukaryotic
Q9FKM2	-	274	Whole sequence (0.977)	Porin, eukaryotic
Q9M2W6	-	226	Whole sequence (0.998)	Porin, eukaryotic
Q9FHQ9	-	163	Whole sequence (0.998)	Porin, eukaryotic
Q8LGE2	-	425	C-terminal 300 (0.569)	Porin, eukaryotic
Q9LHE5	Tom40 homolog 1	309	Whole sequence (0.991)	Porin, eukaryotic
Q9SX55	Tom40 homolog 2	310	C-terminal 300 (0.999)	Porin, eukaryotic
Q8LEH7	-	524	C-terminal 300 (0.986)	Bacterial surface antigen
Q9SRL6	-	520	C-terminal 300 (0.995)	Bacterial surface antigen
Q9LXP7	-	435	Whole sequence (0.645)	Bacterial surface antigen
Q5PP51	-	362	C-terminal 150 (0.900)	Bacterial surface antigen
CBOMP				
Q9C5J8	Toc75-V/OEP80	732	C-terminal 300 (0.746)	Bacterial surface antigen
O80565	OEP37	343	N-terminal 300 (0.863)	-
Q3EBH0	OEP37 homolog	333	C-terminal 300 (0.891)	-
Q3EBG9	OEP37 homolog	280	Whole sequence (0.645)	-
Q1H5C9	OEP24	213	Whole sequence (0.562)	-
A8MR28	OEP24 homolog	167	Whole sequence (0.666)	-
Q9FPG2	OEP21	167	Whole sequence (0.925)	-
Q9LM70	OEP21 homolog	203	C-terminal 150 (0.691)	-
Q6ID99	OEP21 homolog	167	N-terminal 150 (0.875)	-
Uncertain protein				
Q8VYB6	-	491	Whole sequence (0.535)	Protein of unknown function (DUF1005)
Q9LPM5	-	460	C-terminal 450 (0.855)	Protein of unknown function (DUF1005)
Q9M0F0	-	424	N-terminal 300 (0.528)	Protein of unknown function (DUF1005)
Q9LEU1	-	389	N-terminal 300 (0.971)	Plant protein of unknown function (DUF868)
Q9SIS2	-	354	N-terminal 300 (0.655)	Plant protein of unknown function (DUF868)
Q9M903	-	479	C-terminal 150 (0.993)	Protein of unknown function (DUF3769)
O80503	-	451	C-terminal 300 (0.648)	Protein of unknown function (DUF3769)
Q3EAC5	-	134	Whole sequence (0.704)	Domain of unknown function (DUF3406)
Q9SXB7	-	424	C-terminal 300 (0.652)	-
Q9LH72	-	483	C-terminal 300 (0.994)	-
Q9LPG1	-	468	C-terminal 300 (0.816)	-
Q8W4R2	-	271	Whole sequence (0.729)	-
O48573	-	1170	C-terminal 150 (0.625)	-
Q8VY85	-	188	C-terminal 150 (0.608)	-
Q9LZB6	-	177	C-terminal 150 (0.506)	-
Q9M238	-	163	C-terminal 150 (0.748)	-

In summary we found 13 known or probable MBOMPs from known MBOMP families, 9 known CBOMPs, one promising BOMP candidate and 15 unannotated candidates which are either false positives or distant enough from known BOMPs that they elude state of the art sequence-sequence and sequence-structure similarity detectors. Currently we cannot rule out either possibility. Interestingly, one of these 15 has a *β*-signal match, suggesting the possibility that it may be an MBOMP.

Our results differ significantly from an earlier *in silico *study performed by Schleiff et al. [48], in which 891 candidate CBOMP proteins were identified. The Schleiff et al. pipeline used a BOMP predictor developed by Wimley [51] and differs from ours in other ways as well, but the overall scheme is very similar. Yet the number of candidate CBOMP proteins differs by an order magnitude. Clearly both estimates cannot be (even approximately) correct, so this discrepency should be explained if possible.

Although we are unable to give a definitive argument in favor of our estimate, we point out one important difference between these two studies. Schleiff et al. [48] used known CBOMPs to tune their pipeline; they lowered the threshold of their *β*-barrel predictor score from the value of 1.0 recommended by Wimley based on bacterial BOMPs to 0.7, and made other major adjustments in order to include known CBOMPs. In contrast, our study was originally completely focused on mitochondria and thus all the programs and parameters in our pipeline were chosen without considering information about known CBOMPs.

Despite this fact our pipeline detects 9 out of 11 known CBOMP proteins, which suggests that our results are not overly conservative. However our pipeline did miss two identified CBOMPs (Toc75-III and Toc75-IV) and thus may very well have missed some as yet unidentified CBOMPs.

#### Is At1g11320 an novel Arabidopsis BOMP?

Q9SXB7:At1g11320 has 424 residues. The C-terminal 300 residue segment (125-424) was predicted by our predictor to include a *β*-barrel. HHomp detected Cyanobacterial SomB as a At1g11320 homolog (probability score = 100%), and HHpred and FORTE both judged At1g11320 to be similar to bacterial *β*-barrels. HHpred judged the 159-273, 91-232 and 322-424 segments to be similar to the 282-400 segment of *P*. putida toluene transporter TodX (PDBID:3BS0), to 13-141 of *E*. coli OMPA (PDBID:1QJP) and to 141-253 of *N*. meningitides OpcA (PDBID:2VDF), respectively. While FORTE identified similarities between the 134-424 segment of At1g11320 and the 378-865 segment of *S*. marcescens HasR (PDBID:3SCL).

The interpretation of these results is not completely clear, as the predictions involve a patchwork of different bacterial *β*-barrel structures. However, they do suggest the possibility that At1g11320 is a BOMP. It could be either a CBOMP or an MBOMP. The fact that chloroplasts are believed to descend from cyanobacteria and HHomp assigns a high homology probability to a cyanobacterial BOMP, favors the possibility that it is a CBOMP. We also examined the C-terminal region for a *β*-signal match. It does have a match, SDGRTIGL, to the originally proposed consensus of P_o_xGxxH_y_xH_y_, overlapping the last predicted *β*-strand. However, both of the two residues neighboring glycine are hydrophilic and seldom if ever observed in *β*-signals (Table 1), so most likely this is not a genuine *β*-signal.

### Search for novel MBOMPs

Our comprehensive search based on the *β*-signal only detected MBOMPs from known MBOMP families. We also conducted *β*-signal independent searches on the yeast and Arabidopsis proteomes; yielding no new candidates for yeast, but a few for Arabidopsis, one of which seems promising. However it is not yet clear whether this candidate really is an MBOMP.

Recently Remmert et al. [34] performed a bioinformatic proteome analysis using a transitive sequence profile similarity based homology detection method, but only detected four known (two VDAC isoforms, Tom40 and Sam50) and no undiscovered MBOMPs in yeast. Subsequently, these authors showed evidence that all outer membrane barrels from Gram-negative bacteria form a monophyletic group that descended from a single *ββ*-hairpin and suggest this probably is true of MBOMPs as well [52]. Their work supports our hypothesis and complements the search we report here. Their work should be very sensitive at detecting homologs, while our novel MBOMP predictor has the potential to detect analogs or distant homologs with no detectable sequence signal remaining.

However it is important to consider what kind of hypothetical MBOMPs could elude our search. Although not designed for CBOMP detection, when we applied our pipeline (without the *β*-signal filter) to Arabidopsis it detected most known CBOMPs - but missed two. One of these, Toc75-III, has an N-terminal bipartite transit peptide which is cleaved during biogenesis [4]. Of all known Eukaryotic BOMPs, Toc75-III is the only one with a cleavable targeting signal. If such MBOMPs also exist, our search would probably miss them.

Our search might miss multimeric MBOMPs. Several BBOMPs are known to form a *β*-barrel structure from multiple protein chains. For example TolC forms a 12 strand *β*-barrel structure from three TolC molecules, each contributing 4 *β*-strands [53]. Unfortunately TolC, and other multimeric BOMPs, possess large soluble domains in addition to their *β*-barrels, and since each sequence only contains a fraction of the *β*-barrels, the overall fraction of *β*-strand secondary structure is quite small. Our prediction method attempts to account for *β*-barrels with soluble domains through the use of segmentation. However this is not sufficient to detect proteins like TolC, for which the *β*-barrel sequence region is very short. Currently it is not known if any multimeric MBOMPs exist, but if they do we must admit that our pipeline would probably miss them.

Although the currently accepted known MBOMPs all use the *β*-signal for membrane integration, MBOMPs using other mechanisms may exist. If they do, our pipeline would probably miss them. We only applied the *β*-signal independent version of our search on two model organisms. Ideally we would like to apply it to all Eukaryotic proteins, but unfortunately that would result in a list of hundreds or thousands of candidate proteins, without much annotation or additional information to cull or prioritize the list. Even when applied to Arabidopsis, our *β*-signal independent produced 15 candidate BOMP proteins, for which we can not find corroborating evidence to support or refute. From our results, we can conclude that in the yeast proteome, it is unlikely that we missed any novel undiscovered MBOMP simply because they lack *β*-signals, but rather they would have to differ more dramatically from known MBOMPs. Unfortunately we can make no strong conclusions regarding the possibility of *β*-signal independent MBOMPs in other species.

Last, but not least, our search would definitely miss any new MBOMPs which only occur in singleton clusters when clustered at 40% identity. Unfortunately this limitation cannot currently be avoided. As can be seen by the *β*-signal independent Aradopsis search, which yielded at least 22 but perhaps as many as 40 false positives, our method depends on an effective *β*-signal filter to have enough discriminative power to be applied on a Eukaryote-wide scale. Unfortunately the *β*-signal is an information poor motif and occurs quite frequently in non-MBOMP proteins. So the only way to make the *β*-signal filter effectively reduce false positives is to require evidence of conservation, and thus we were forced to discard singleton clusters. It is difficult to "prove a negative" and it will always be possible to suggest that an informatics search of the type performed here may miss MBOMPs due to incomplete data or incorrect assumptions. However we believe the search describe here, in conjunction with the consistent results of Remmert et al. [34] obtained with a different methodology, offer strong enough evidence to place the burden of proof on anyone who advocates the existence of undiscovered MBOMP families. Two potential avenues to pursue this are refined proteomics techniques which can distinguish between BOMP and other outer membrane proteins, and sequence analysis of novel organisms not yet included in Uniprot.

#### Uth1 not an MBOMP?

We mentioned yeast Uth1 as the only candidate MBOMP identified in our preliminary work [23]. However, further analysis suggests that Uth1 is probably not an MBOMP. Uth1 has a *β*-signal motif match near its last predicted *β*-strand, however our new *β*-signal independent MBOMP predictor does not predict it to be an MBOMP.

Uth1 was reported to have an outer membrane localization [54], but the authors did not specifically claim that it is a *β*-barrel. Another study failed to confirm its mitochondrial localization, "Preliminary yeast cell fractionation analyses showed that Uth1 with an epitope tag was not efficiently recovered with mitochondria, suggesting that it is not a mitochondrial protein" (Yamano, K. and Endo, T., personal communication). Thus, we conclude that Uth1 probably is not an MBOMP.

### *β*-signal

Our MBOMP homolog analysis confirms and slightly refines the *β*-signal. As can be inferred from the statistics in Table 1 and 2; in Mdm10 the first position is not always polar, but glycine and other small residues are sometimes seen. From Table 1 we can also see that in the homologs we examined, the fifth position never contains a large hydrophobic residue. From the statistics of Table 1 two patterns with good coverage and *p*-values are $\bar{{\text{H}}_{\text{y}}}{\text{H}}_{\text{y}}{\text{Gh}}_{\text{y}}{\text{xH}}_{\text{y}}{\text{xH}}_{\text{y}}$ and $\bar{{\text{H}}_{\text{y}}}{\text{H}}_{\text{y}}{\text{Gh}}_{\text{y}}\bar{{\text{H}}_{\text{y}}}{\text{H}}_{\text{y}}{\text{xH}}_{\text{y}}$. The *p*-values for these two patterns are nearly the same for the background sequence regions from MBOMP proteins used to compute them. However we would expect the more specific pattern $\bar{{\text{H}}_{\text{y}}}{\text{H}}_{\text{y}}{\text{Gh}}_{\text{y}}\bar{{\text{H}}_{\text{y}}}{\text{H}}_{\text{y}}{\text{xH}}_{\text{y}}$ to discriminate better when non *β*-barrel sequences are included in the background. In that case a requirement of no prolines could also be considered as an extra filter, or alternatively a position weight matrix covering these positions could be defined.

Amongst the putative MBOMP homologs, we found 13 exceptions to the core *β*-signal consensus, and 12 of them were in Mdm10. Mdm10 also differs from the other MBOMP families in that it has no known homologs in animals.

Even the exceptions contain the apparently invariant glycine and exhibit the length 2 periodicity expected in a *β*-strand. However, our stastical analysis of the frequency of *β*-signal motifs in the C-terminal versus other parts of MBOMP sequences (Table 2) suggest that the non-glycine positions of the *β*-signal may contribute to its recognition in a more specific way than just by providing an appropriate secondary structure context.

Recent experimental work suggests that the *β*-signal is not necessary for mitochondrial outer membrane integration. Walther et al. [36] reported that, when expressed in yeast, some BBOMPs were sorted to the mitochondrial outer membrane. In particular the C-terminus of OMP85_NEIMB does not even have a near match to the *β*-signal (Figure 5). It remains to be seen if any naturally occurring MBOMPs lack a *β*-signal. As discussed above, in a Eukaryote-wide search, removing the *β*-signal filter leads to an unmanageable number of candidates. One idea for future work, would be to use a bacterial-type C-terminal phenylalanine integration signal motif [55] as an alternative filter.

## Conclusions

We developed a novel MBOMP predictor, and used it to perform a Eukaryotic-wide search for *β*-signal dependent MBOMPs, and a *β*-signal independent search in yeast and Arabidopsis. In the Eukaryotic-wide search we found no promising new MBOMP candidates. Likewise in the yeast *β*-signal independent search we found no promising new MBOMP candidates. The results of the *β*-signal independent Arabidopsis search were less conclusive. We found one potentially promising new BOMP candidate and 15 more candidate BOMPs which do not appear to have sequence or structural similarities to known BOMPs. We could not find corroborating evidence to support these candidates, so their confirmation or refutation must await further work.

We conclude that the currently identified MBOMP protein families may represent a nearly complete repertoire of *β*-signal dependent MBOMPs. If many undiscovered MBOMP families remain in the current sequence databases, they most likely differ from known MBOMPs significantly in terms of structure or biogenesis.

## Methods

### Known MBOMP ortholog sets

Using Swiss-Prot 57.8 [56], we gathered all complete protein sequences with at least 40% identity to known MBOMPs, a level which strongly suggests similar structure. After removing nearly identical sequence pairs we obtained a set of 70 proteins in which each pair shares less than 90% sequence identity. The expression of 46 (VDAC:16, Tom40:8, Sam50:6 and Mdm10:16) of these has been confirmed at the protein or transcript level.

### Search for novel MBOMPs in eukaryotic proteomes

#### Eukaryotic Protein Sequence Cluster Dataset

We downloaded all eukaryotic protein sequences in Uniprot Version 15.1 and applied a simple filter to obtain 1,238,639 protein sequences with no more than 95% sequence identity. We then clustered these sequences at 40% identity with BLASTClust http://blast.ncbi.nlm.nih.gov/ and removed singleton clusters from further consideration. In this way we obtained 105,547 non-singleton clusters, containing a median of 3 and average of 8.8 sequences per cluster.

We computed a multiple alignment of each cluster with ClustalW [57]. Given the 40% sequence identity level within clusters, we expect the sequences should be relatively easy to align correctly.

#### Search Procedure

We searched intensively for novel MBOMPs, relaxing the constraint imposed in our earlier work, of requiring a C-terminal location of potential *β*-signals. Figure 3 shows the pipeline we employed to identify proteins with either C-terminal (C-terminal 40 residues region) or internal (whole sequence except for the C-terminal 40 residues) potential *β*-signals. Where a potential *β*-signal is a match to the *β*-signal motif in each sequence in a cluster, which aligns perfectly in their multiple sequence alignment.

The pipeline is comprised of 6 filters:

• **Potential ***β***-signal: **a sequence passes this test if it has an appropriately placed match to the *β*-signal motif (P_o_xGh_y_xH_y_xH_y_) which aligns perfectly in its cluster multiple alignment.

• **Not an α-helical membrane protein: **a sequence fails this test if it is predicted to be an α-helical membrane protein by either Phobius [58] or TMHMM [59].

• **Not a secretory path protein: **a sequence fails this test if it is predicted to have a signal peptide by either TargetP or Phobius.

• **No MTS: **a sequence fails this test if it is predicted to have a mitochondrial matrix targeting signal (MTS) by either TargetP [60] or Predotar [61].

• **Not an ***α***-rich protein: **a sequence fails this test if it is predicted by PSIPRED [46] to have less than 25% *β*-strand and more than 10% α-helical secondary structure content in the (up to 300) residues preceding the *β*-signal.

• *β***-signal match not of ***α ***-helical structure: **fails test if more than 50% of the 8 residues of *β*-signal match are predicted as *α *-helical structure by PSIPRED.

• **Manual inspection: **annotation of subcellular localization, family and domain by Gene ontology, Pfam [62] and interPro [43].

• **MBOMP predictor: **fails test if not predicted to be an MBOMP by our MBOMP predictor (not applied to C-terminal *β*-motif matches).

### MBOMP Predictor

We trained a novel MBOMP predictor on known MBOMP sequences, but with some features derived from BBOMP structures. We chose not to use existing tools because they are trained only on bacterial sequences. Instead we trained our MBOMP predictor on a mixture of bacterial and mitochondrial proteins. The features used by our MBOMP predictor are: three types of amino acid composition (standard, bigram and gapped bigram); physicochemical features of the N-terminal sorting region, periodic amphiphilicity scores and position weight matrices (computed from BBOMP structures) to model transmembrane *β*-strands and 2-strand *ββ*-hairpins (Figure 6). For a classifier, we chose a support vector machine (SVM) with an RBF kernel, as implemented in the LIBSVM package http://www.csie.ntu.edu.tw/~cjlin/libsvm. All features were linearly scaled to the range [-1,1] before being presented to the SVM classifier.

#### Features

##### Length

We do not use length as a feature per se, but we require candidate MBOMPs to be at least 90 residues. This should not exclude single chain barrels, as even the most compact barrels span about 150 residues [63].

##### Amino Acid Composition

It is well known that protein secondary structure correlates with amino acid composition and invokes correlation between nearby residues [64]. Therefore we adopted amino acid composition, dipeptide composition and "skip one dipeptide composition", defined as the frequency of the 400 patterns A-X-B, where X is any residue and A and B are fixed amino acid residues, to give a total of 820 features.

##### Signal Peptide Related Features

**Figure 7:**
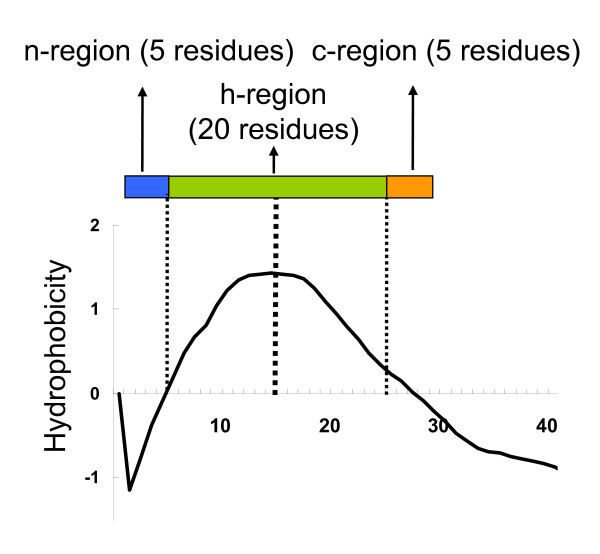
**Signal Peptide Model**. The h-region is defined as the region of 20 residues around the position of the first peak of the hydrophobicity plot, and the n-region and c-region as the N-terminal and C-terminal flanking 5 residues, respectively, as shown at the top of this figure. The h-region is aligned to the first peak of the hydrophobicity plot (details in main text).

MBOMPs are not expected to have secretory pathway signal peptides. We therefore included four signal peptide related features. Following the classical model of signal peptides [65], we defined features according a contiguous block of 30 residues, partitioned into the n-, c-, and h-regions, of length 5, 20 and 5 residues respectively (Figure 7). Denoting the Kyte-Doolittle hydrophobicity [66] of the *i*th residue as *h*(*i*), we compute a weighted average of the hydrophobicity near residue *j *as:

(1) $$H\left(j\right)=\sum_{i=j-14}^{j+14}(15-|i-j|)*h(i)/15^{2}$$

We define the N-terminal peak position as the first peak attaining at least 0.6, up to the first 70 residues, or the maximum if no such peak exists. More precisely the peak position is arg max_*j*ϵ*R *_*H*(*j*), where *R *is either the first contiguous set of positions in the interval [1, 70] such that *H*(*j*) ≥ 0.6; or if that set is empty, *R *is simply the first 70 positions.

When computing features, we aligned the 10th position of the h-region to the N-terminal peak. We used the following features:

(2) $$\begin{array}{l} 1)\sum_{i\;\in \text{ h-region}}h(i),\;2)\sum_{i\;\in \text{ n-region}}p(i),\;3)\sum_{i\;\in \text{ n-region}}n(i),\\ \;4)\sum_{i\;\in \text{ n-region}}n(i)\;\text{and}\;\text{5)}\sum_{i\;\in \text{ c-region}}o(i) \end{array}$$

where *p*(*i*), *n*(*i*) and *o*(*i*) are 0-1 binary variables equal to 1 when the *i*th residue is positively {K, R, H} or negatively {D, E} charged, or one of {A, G, S}, respectively.

##### Amphiphilicity scores

Since every other residue in a BOMP *β*-strand tends to be hydrophobic, we define a tile amphiphilicity score:

(3) $$A\left(j,l\right)=\left|\sum_{i\in \left\{0,2,...l\right\}}h\left(j+i\right)-\sum_{i\in \left\{1,3,...l-1\right\}}h(j+i)\right|$$

for the length *l *tile starting at position *j*.

Let *A*(*R, l*) denote the 60th percentile of *A*(*j*, *l*) for *j *in sequence region *R*, and *n *denote the entire sequence length. To account for the fact that BOMPs contain a significant number of residues in loops and often non-barrel domains as well, we defined three types of region amphiphilicity scores: global, *A*(*R*, *l*) where *R *= [1, *n*]; N-terminal, *R *= max_*Q*ϵ{[1, 90], [1, 91],...[1,*n*]} _*A*(*Q*, *l*); and C-terminal,

*R *= max_*Q*ϵ{[*n*-89,*n*],[*n*-90,*n*],...[1,*n*]} _*A*(*Q*, *l*). The N- and C-terminal features are designed to detect proteins with N- or C-terminal barrel domains respectively. Finally we define 9 amphiphilicity related features to show to the classifier: each of the three region amphiphilicity scores computed with tile lengths of 8, 10 and 12.

##### PWMs for transmembrane *β*-strands and *ββ*-hairpins

We collected 42 BBOMP structures with less than 40% sequence identity from PDB [67]. From these structures we extracted 428 transmembrane segments of lengths 8, 9 and 10; and 181 *ββ *-hairpins (periplasmic short loop plus 8 residues on both sides of the loop) with loop lengths of 3, 4 and 5. As extracted, the *β*-strands contained in these segments were not always in phase with each other in terms of amphiphilicity. Thus we first expressed each segment as a vector of hydrophobicity values *h*(*i*), and used those vectors to divide each segment into one of two clusters using the hierarchical clustering function of the R programming language with Euclidean distance. For the hairpin types we defined a PWM for each cluster, yielding 2 PWMs each, of lengths 19, 20 and 21. The initial PWMs derived from each pair of clusters of the single strand transmembrane segments were nearly identical except for being shifted by one position relative to each other. Thus we combined these PWMs by shifting one of them by one position relative to the other and discarding the unmatched columns at each end. This yielded 3 PWMs of lengths 7, 8 and 9, respectively. For a background model we used the overall amino acid composition of sequences in our dataset.

To convert a PWM *P*, into a numerical feature, we first compute *r*, the maximum log likelihood ratio score between *P *and the background model for each possible position in the query sequence. More precisely

(4) $$\begin{array}{c} r=\overset{n-w+1}{\underset{j=1}{\max}}r\left(j\right)\\ r\left(j\right)=\sum_{i=1}^{w}\frac{P(s\left[i+j-1\right],i)}{B(s\left[i+j-1\right]} \end{array}$$

Where *w *denotes the width of the PWM, and *s*[*i*] the *i*th character in the query sequence. In general *r *will tend to increase with sequence length and we speculate that it will follow an extreme value distribution similar to the BLAST statistics [68]. Using non-mitochondrial sequences, we empirically confirmed that the following normalization effectively removes the dependence on sequence length for background sequences (results not shown).

(5) $$r^{\prime }=r-a*\log(n)$$

where *a *is an empirically adjusted constant which differs for each PWM. Thus we defined the nine PWM based features with that formula.

#### MBOMP Predictor Dataset

Our dataset contains 94 positive examples, consisting of the known yeast MBOMPs and their presumed (based on sequence similarity and conservation of the *β*-signal) homologs in other species. No positive example shares more than 40% identity with any other. For negative examples we used 2640 yeast proteins with clear annotation of subcellular localization of Uniprot and less that 25% mutual sequence identity.

#### MBOMP Predictor Results

To assess the accuracy of our MBOMP predictor, we measured the performance with 5-fold cross validation. We obtained a precision of 98.8 ± 0.03%, recall of 84.2 ± 0.06% and F-measure of 0.91 ± 0.04% (Table 5). To further test the ability of the predictor to generalize to novel MBOMP families, we trained it using only homologs of {VDAC, Tom40, Sam50} as MBOMP positive examples (BBOMPs were also included as positive examples in all cases), and then tested whether this predictor could correctly classify Mdm10 (as positive) and Mmm2 family (as negative). We believe this is a good test, because Mdm10 is a bit different from the other MBOMPs (it has not been found in animals and its *β*-signal seems a bit divergent), while Mmm2 is plausible enough as an MBOMP that it was considered to be one for some time. In this test, our predictor predicted nine of twelve Mdm10 homologs and zero of eleven Mmm2 homologs.

**Table 5:** Prediction performance of our SVM MBOMP predictor.

Fold	TP	FP	FN	TN	Precision	Recall	Specificity	F-measure
1	15	1	4	527	0.938	0.789	0.998	0.857
2	16	0	3	528	1.000	0.789	1.000	0.914
3	15	0	4	528	1.000	0.789	1.000	0.882
4	16	0	3	528	1.000	0.842	1.000	0.914
5	17	0	1	528	1.000	0.944	1.000	0.971
Mean	-	-	-	-	0.988	0.842	1.000	0.908

### *β*-signal independent search in yeast and Arabidopsis proteomes

We searched for MBOMPs lacking *β*-signals in 6470 *S*. cerevisiae and 35555 *A*. thaliana proteins found in Uniprot by combining our MBOMP predictor with PSIPRED secondary structure prediction, Phobius *α*-helical membrane protein and signal peptide prediction and finally manual inspection based on annotation. In this search, in addition to whole sequences, we considered N-terminal and C-terminal protein segments of length 150, 300 and 450 residues, to take into account the possibility of multi-domain MBOMPs such as Sam50 (Additional file 3, Figure S3); thus proteins with sufficiently long amino acid sequences were input in seven sequence forms and considered an MBOMP if any of those were predicted positive. The lengths of 150 and 450 were chosen to cover the size of most *β*-barrel domains in BOMPs of known structure.

In the secondary structure prediction and manual inspection step, we disregarded any proteins or segments with more than 10% *α*-helical and less than 25% *β*-strand predicted secondary structure content and with the annotation of subcellular localization, family and domain, respectively.

### Sequence Logos

For each ortholog set, we perfomed multiple alignment with ClustalW [57] and extracted the octomer region corresponding to the *β*-signal region proposed by Kutik et al [35]. From this set we removed identical octomers, yielding 57 octomers from the original 70 and 41 octomers from the expression confirmed set. We designed this procedure to trade off the desire to use as much data as possible, with the risk of creating biased results by including too many variations of highly similar sequences. Since the sequences are not all mutually independent, the heights of the columns are only suggestive, but the general trends remain the same when a sequence identity cutoff of 30% is employed to reduce sequence interdependence (Additional file 4, Figure S4).

## Authors' contributions

KI performed most of the experiments and prepared much of the manuscript. KI, NF and PH developed the novel MPOMP predictor. MG helped plan the study and refine the manuscript. PH conceived the study and wrote some of the manuscript. All authors read and approved the final manuscript.

## Supplementary Material

Additional file 1 **Figure S1 - Multiple alignment of N-terminal region of putative Sam50 proteins**. Multiple alignment and secondary structure prediction (by PSIPRED; orange: α-sheet, blue: β-sheet) of the N-terminal region of 9 proteins containing the bacterial surface antigen (POTRA) domain are shown. Click here for file

Additional file 2 **Figure S2 - β-signal motif match in YJL217W**. (a) Secondary structure prediction (by PSIPRED) of the uncharacterized protein YJL217W and its match to the *β*-signal motif is shown. (b) 3 D structure of YJL217W (PDBID:3O12) Click here for file

Additional file 3 **Figure S3 - Sequence segmentation**. The sequence segmentation used to search for, possible multiple domain, yeast MBOMPs with our SVM-predictor is shown. Click here for file

Additional file 4 **Figure S4 - Sequence logo of β*-signal***. Sequence logos are displayed for 70 MBOMP homologs which remain after omitting redundant sequences with more than 30% identity. Click here for file
