# Supplementary material for: Eukaryote-wide sequence analysis of mitochondrial β-barrel outer membrane proteins
Source: BMC Genomics. 2011 Jan 28;12:79. doi: 10.1186/1471-2164-12-79 (PMC3045335; doi:10.1186/1471-2164-12-79)
Supplement: Additional file 1 — Figure S1 - Multiple alignment of N-terminal region of putative Sam50 proteins. Multiple alignment and secondary structure prediction (by PSIPRED; orange: α-sheet, blue: β-sheet) of the N-terminal region of 9 proteins containing the bacterial surface antigen (POTRA) domain are shown. [file 1471-2164-12-79-S1.PDF]

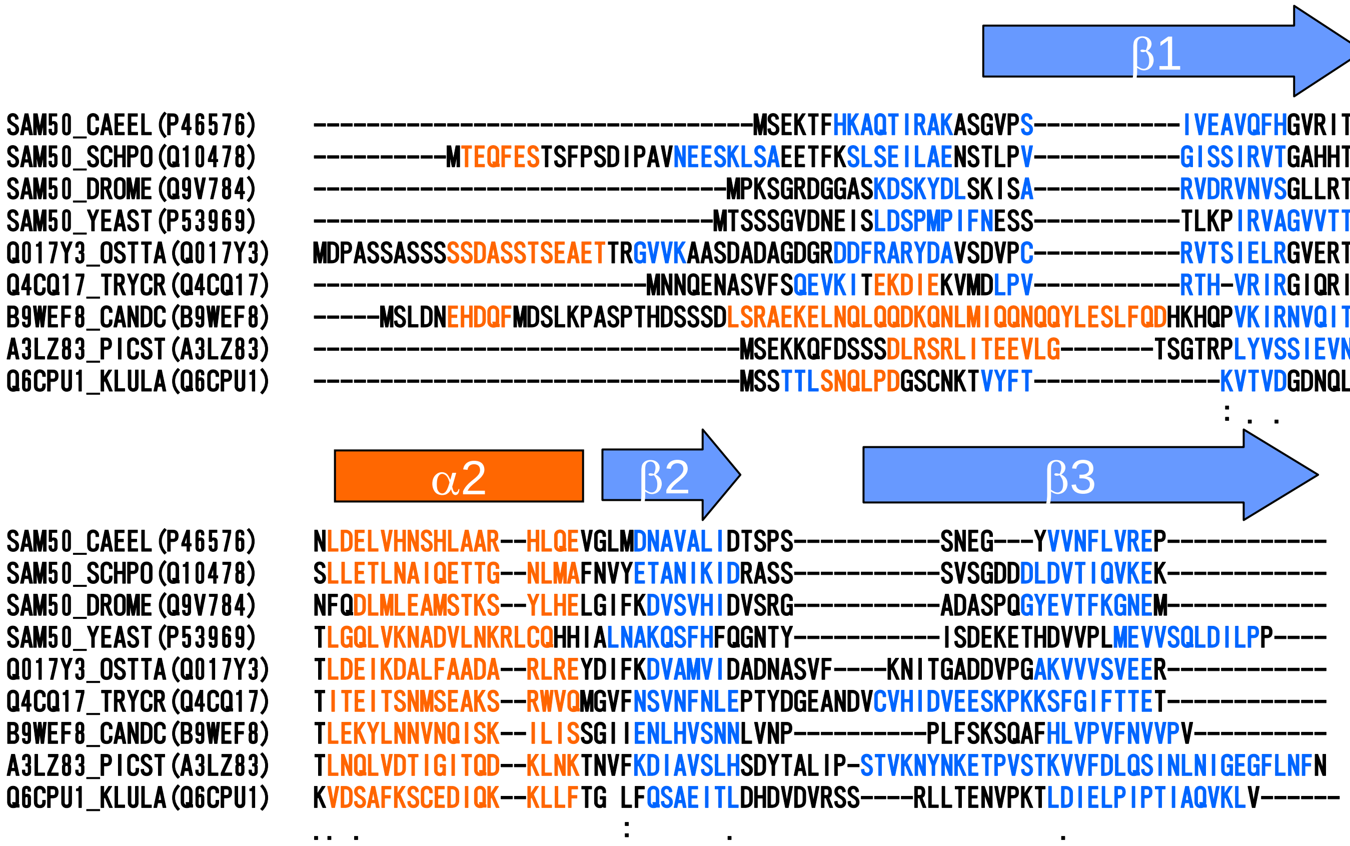
β1
α1
SAM50_CAEEL (P46576) ---------------------------------MSEKTFHKAQTIRAKASGVPS-----------IVEAVQFHGVRITKN----DALVK---EVSELYRSK
SAM50_SCHPO (Q10478) ----------MTEQFESTSFPSDIPAVNEESKLSAEETFKSLSEILAENSTLPV-----------GISSIRVTGAHHTRPS-FIRKVLKTCLDTSKPAKSR
SAM50_DROME (Q9V784) -------------------------------MPKSGRDGGASKDSKYDLSKISA-----------RVDRVNVSGLLRTHN----DYVMR---AADGLFKAS
SAM50_YEAST (P53969) ------------------------------MTSSSGVDNEISLDSPMPIFNESS-----------TLKPIRVAGVVTTGTD-HIDPSVLQAYLDDTIMKSI
Q017Y3_OSTTA (Q017Y3) MDPASSASSSSSDASSTSEAETTRGVVKAASDADAGDGRDDFRARYDAVSDVPC-----------RVTSIELRGVERTRV----ELIER---EMERARRAK
Q4CQ17_TRYCR (Q4CQ17) -------------------------MNNQENASVFSQEVKITEKDIEKVMDLPV-----------RTH-VRIRGIQRIHP----RVISR---DVEAIKRSR
B9WEF8_CANDC (B9WEF8) -----MSLDNEHDQFMDSLKPASPTHDSSSDLSRAEKELNQLQQDKQNLMIQQNQQYLESLFQDHKHQPVKIRNVQITNGQSYRDQFLKYQFRNLLNGEVM
A3LZ83_PICST (A3LZ83) --------------------------------MSEKKQFDSSSDLRSRLITEEVLG-------TSGTRPLYVSSIEVNGGETFSAEFFKKLLTPLIDRSDY
Q6CPU1_KLULA (Q6CPU1) --------------------------------MSSTTLSNQLPDGSCNKTVYFT--------------KVTVDGDNQLSP--VSDDLYQSIFNRILADPLQ
: . .
α2
β2
β3
SAM50_CAEEL (P46576) NLDELVHNSHLAAR--HLQEVGLMDNAVALIDTSPS-----------SNEG---YVVNFLVREP------------
SAM50_SCHPO (Q10478) SLLETLNAIQETTG--NLMAFNVYETANIKIDRASS-----------SVSGDDDLDVTIQVKEK------------
SAM50_DROME (Q9V784) NFQDLMLEAMSTKS--YLHELGIFKDVSVHIDVSRG-----------ADASPQGYEVTFKGNEM------------
SAM50_YEAST (P53969) TLGQLVKNADVLNKRLCQHHIALNAKQSFHFQGNTY-----------ISDEKETHDVVPLMEVVSQLDILPP----
Q017Y3_OSTTA (Q017Y3) TLDEIKDALFAADA--RLREYDIFKDVAMVIDADNASVF----KNITGADDVPGAKVVVSVEER------------
Q4CQ17_TRYCR (Q4CQ17) TITEITSNMSEAKS--RWVQMGVFNSVNFNLEPTYDGEANDVCVHIDVEESKPKKSFGIFTTET------------
B9WEF8_CANDC (B9WEF8) TLEKYLNNVNQISK--ILISSGIIENLHVSNNLVNP----------PLFSKSQAFHLVPVFNVVPV----------
A3LZ83_PICST (A3LZ83) TLNQLVDTIGITQD--KLNKTNVFKDIAVSLHSDYTALIP-STVKNYNKETPVSTKVVFDLQSINLNIGEGFLNFN
Q6CPU1_KLULA (Q6CPU1) KVDSAFKSCEDIQK--KLLFTG LFQSAEITLDHDVDVRSS----RLLTENVPKTLDIELPIPTIAQVKLV------
.. . : . .
