# Supplementary material for: Eukaryote-wide sequence analysis of mitochondrial β-barrel outer membrane proteins
Source: BMC Genomics. 2011 Jan 28;12:79. doi: 10.1186/1471-2164-12-79 (PMC3045335; doi:10.1186/1471-2164-12-79)

(a)

P40893: Uncharacterized protein YJL217W

CCCCCCCCHHHCEEECCCCEEEEECCEEEEEECCCCCEEECCCCCCEECCCCEEEEECCCCEEEEEEEECCCCEEEEEEE
MVESKNTELSQGTWLNKPKSVFQEAGKVTLETDEKTDFWRETFYGFTRDSGHFLGVETGSAFTAQVRVQGSYESLYDQAG

EEEEECCCCEEEEEEEEECCCEEEEEEECCCCCCCCCCCCCCCCCEEEEEEEEECCEEEEEEECCCCEEEEEEEEECCCC
IMVRIDDGHWLKAGIEISDGHAMLSSVLTNGKSDWSTAVYGGNARDFWLRVTVEKGVLRIQVSSDKKTWPLVRLAPFPTS

CEEEEEEEEECCCCCCEEEEEEEEEECCCCCHHCCCCC
DHYLVGPMACTPERGGLKVTFSEWSLTAPLGKALHDLS

β-signal motif

(b)

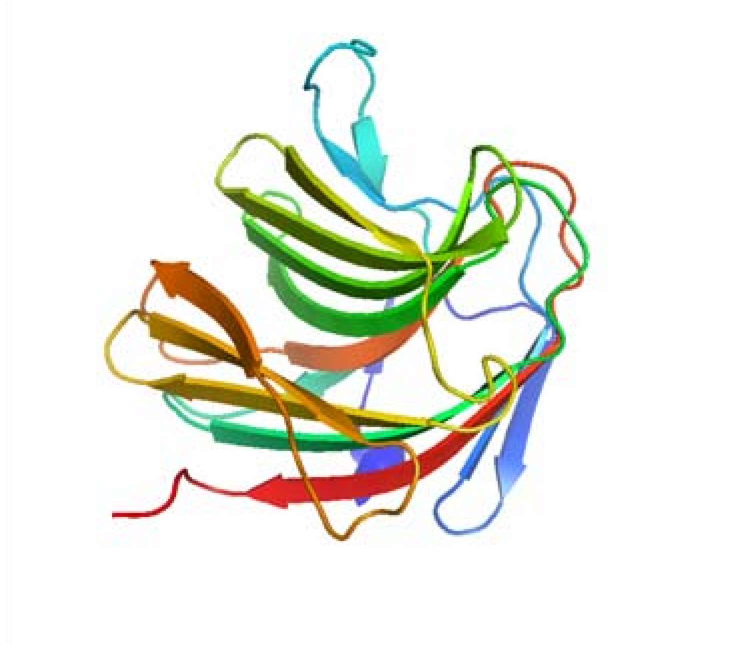

Supplement: Additional file 2 — Figure S2 - β-signal motif match in YJL217W. (a) Secondary structure prediction (by PSIPRED) of the uncharacterized protein YJL217W and its match to the β-signal motif is shown. (b) 3 D structure of YJL217W (PDBID:3O12) [file 1471-2164-12-79-S2.PDF]
