# Supplementary material for: Eukaryote-wide sequence analysis of mitochondrial β-barrel outer membrane proteins
Source: BMC Genomics. 2011 Jan 28;12:79. doi: 10.1186/1471-2164-12-79 (PMC3045335; doi:10.1186/1471-2164-12-79)

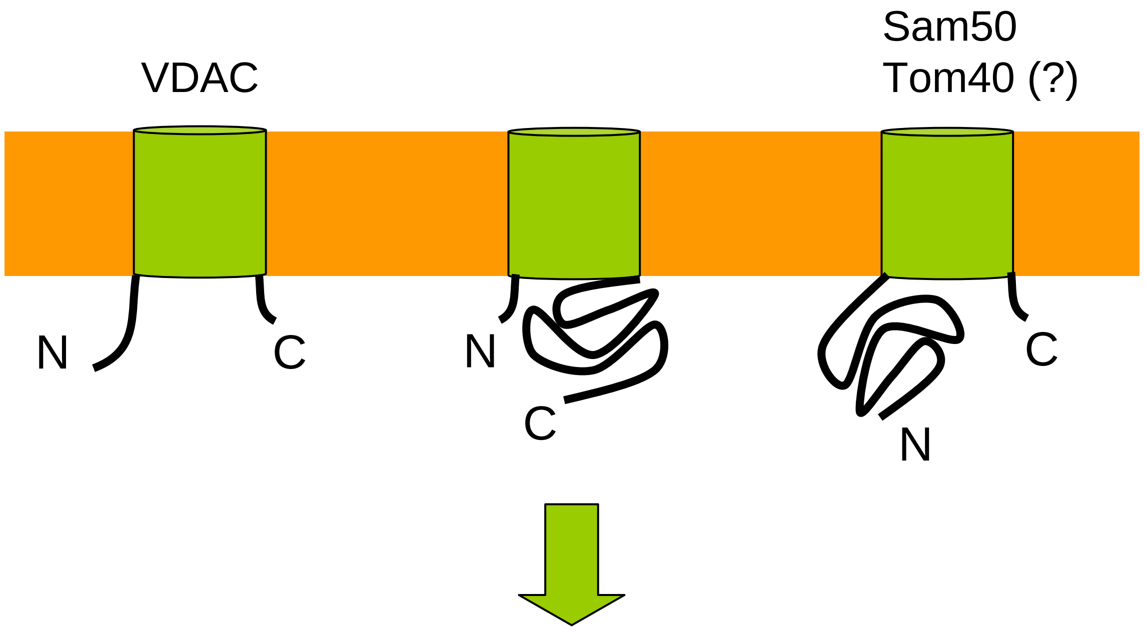

Segmentation of yeast Proteins

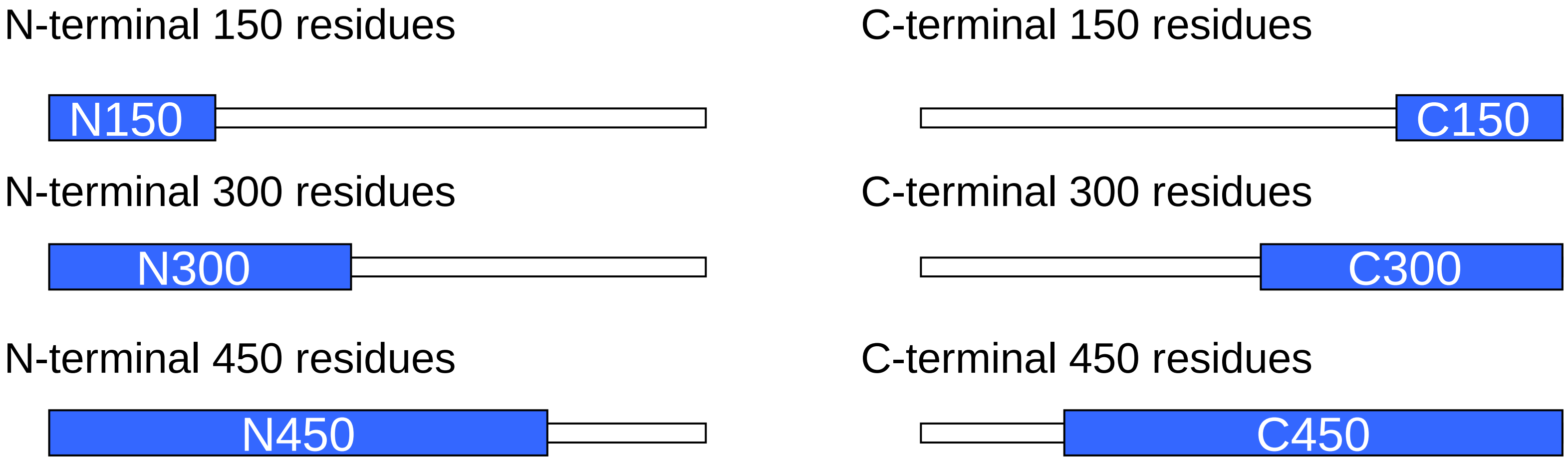

Supplement: Additional file 3 — Figure S3 - Sequence segmentation. The sequence segmentation used to search for, possible multiple domain, yeast MBOMPs with our SVM-predictor is shown. [file 1471-2164-12-79-S3.PDF]
