# Supplementary figures and images for: Eukaryote-wide sequence analysis of mitochondrial β-barrel outer membrane proteins
Source: BMC Genomics. 2011 Jan 28;12:79. doi: 10.1186/1471-2164-12-79 (PMC3045335; doi:10.1186/1471-2164-12-79)

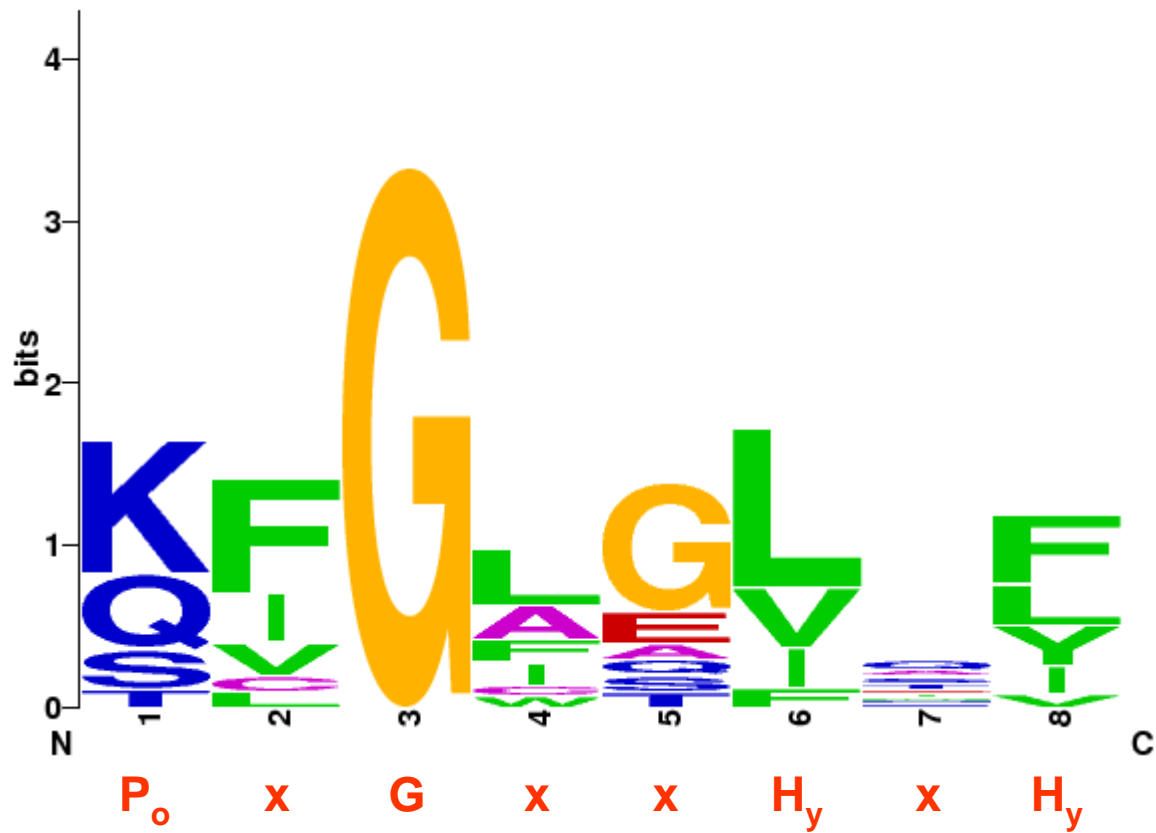

Supplement: Additional file 4 — Figure S4 - Sequence logo of β-signal. Sequence logos are displayed for 70 MBOMP homologs which remain after omitting redundant sequences with more than 30% identity. [file 1471-2164-12-79-S4.PDF]
